# Structural and Metabolic Profiling of *Lycopersicon esculentum* Rhizosphere Microbiota Artificially Exposed at Commonly Used Non-Steroidal Anti-Inflammatory Drugs

**DOI:** 10.3390/microorganisms10020254

**Published:** 2022-01-24

**Authors:** Emoke Dalma Kovacs, Luminita Silaghi-Dumitrescu, Cecilia Roman, Di Tian

**Affiliations:** 1 Research Institute for Analytical Instrumentation, INCDO-INOE 2000, 400293 Cluj-Napoca, Romania; cici_roman@yahoo.com; 2 Faculty of Chemistry and Chemical Engineering, Babes-Bolyai University, 400028 Cluj-Napoca, Romania; luminita.silaghi@gmail.com; 3 Research Center of Forest Management Engineering of State Forestry and Grassland Administration, College of Forestry, Beijing Forestry University, Beijing 100083, China; tiandi@bjfu.edu.cn

**Keywords:** microorganisms, pharmaceuticals, exposure, functioning

## Abstract

In this study, the effect of common non-steroidal anti-inflammatory drugs on *Lycopersicon esculentum* rhizosphere microbiota was monitored. The experiments were performed with artificially contaminated soil with ibuprofen (0.5 mg·kg^−1^), ketoprofen (0.2 mg·kg^−1^) and diclofenac (0.7 mg·kg^−1^). The results evidenced that the rhizosphere microbiota abundance decreased especially under exposure to diclofenac (187–201 nmol·g^−1^ dry weight soil) and ibuprofen (166–183 nmol·g^−1^ dry weight soil) if compared with control (185–240 nmol·g^−1^ dry weight soil), while the fungal/bacteria ratio changed significantly with exposure to diclofenac (<27%) and ketoprofen (<18%). Compared with control samples, the average amount of the ratio of Gram-negative/Gram-positive bacteria was higher in rhizosphere soil contaminated with ibuprofen (>25%) and lower in the case of diclofenac (<46%) contamination. Carbon source consumption increased with the time of assay in case of the control samples (23%) and those contaminated with diclofenac (8%). This suggests that rhizosphere microbiota under contamination with diclofenac consume a higher amount of carbon, but they do not consume a larger variety of its sources. In the case of contamination with ibuprofen and ketoprofen, the consumption of carbon source presents a decreasing tendency after day 30 of the assay. Rhizosphere microbiota emitting volatile organic compounds were also monitored. Volatile compounds belonging to alcohol, aromatic compounds, ketone, terpene, organic acids, aldehyde, sulphur compounds, esters, alkane, nitrogen compounds, alkene and furans were detected in rhizosphere soil samples. Among these, terpene, ketone, alcohol, aromatic compounds, organic acids and alkane were the most abundant compound classes (>75%), but their percentage changed with exposure to diclofenac, ketoprofen and ibuprofen. Such changes in abundance, structure and the metabolic activity of *Lycopersicon esculentum* rhizosphere microbiota under exposure to common non-steroidal anti-inflammatory drugs suggest that there is a probability to also change the ecosystem services provided by rhizosphere microbiota.

## 1. Introduction

Diclofenac, ibuprofen and ketoprofen are common non-steroidal anti-inflammatory drugs (NSAIDs) that are often reported in environmental assessment studies_._ This is because of their high consumption rate [1,2] and improper removal during wastewater treatment processes [3,4]. They reach the soil system either through the reuse of treated municipal wastewater in irrigation purposes [5,6], or from the resulting sludge and biosolids as fertilizers [7,8]. Their common occurrence in soil environments raised high concerns because of the continuous input [9], subsequent accumulation potential [5] and their potential ecotoxicological effects on nontargeted living organisms at different trophic levels [10,11].

Soil microbiota resistance to pharmaceuticals is a global issue and understanding its functional and molecular basis is essential. Bacterial resistance to pharmaceuticals could be either natural or acquired [12]. Although a natural resistance could be not considered a serious clinical issue, the acquired resistance is a much more severe case when we consider the high ability of bacteria to capture fragments of DNA and genes of resistance to many commonly used pharmaceuticals, even from phylogenetically distinct organisms. Through horizontal gene transfer and cross-resistance development, bacteria could become resistant to multiple drugs [13].

Soil microbiota are key actors in soil processes, contributing significantly to numerous ecosystem services provided by soil. They are involved in the processes of nutrient cycling and organic matter degradation [14]. Microorganisms are also able to synthesize volatile organic compounds, such as alcohols, terpenes, ketones, alkanes, etc. [15]. These are secondary metabolites with multiple ecological roles and mechanisms of action. Reports have started to highlight that those volatile organic compounds emitted by soil microbiota can act as signalling molecules assuring distance communication between various organisms [16], can induce inhibitory activity against fungal spore germination [17], those changing microbiota structure, and can modulate enzyme activity [18]. In this way, they can directly influence the aboveground biodiversity and productivity. Through the chemicals that the microorganism cycles or releases, they can stimulate or inhibit plant development [16]. Rhizosphere, the interface between plant roots and soil is one of the most abundant and dynamic system inhabited by microorganism [19]. Rhizosphere microbiomes differ from soil microbiomes. Although it is well acknowledged that the microbiota of rhizosphere have positive effects on plant development and heath [20,21], there is less knowledge on their structure, abundance and function under challenging conditions. Considering the frequency of NSAIDs’ presence in soil environment [1,7,8], studies are essential to unravel the functions of rhizosphere microbiota under exposure to frequently detected NSAIDs.

A major goal in ecology is to assure the development of more stable agrosystems that can face current challenges. This could be achieved through the achievement of a deeper knowledge on rhizosphere ecology and the identification of rhizobiome chemical and biological diagnostics and signatures for identified issues. Microbiota are characterized as small organisms; therefore, compared to other organisms, they present a high surface area-to-volume ratio, which provides a large contact interface that interacts with their surrounding environment, respectively, with surrounding contaminants [22]. Under the exposure to pharmaceuticals, soil microbiome biological parameters such as structure, abundance and metabolic activity could suffer changes. Wang et al. [23] assessed the toxic effects of enrofloxacin on soil enzymatic activities and showed that the activities of sucrase was inhibited significantly at all incubation periods. In studies on microbial utilisation in the Biolog plates, they reported that the utilisation of metabolites was inhibited severely and reached essentially zero after 21 days, although they gradually decreased with the increasing of time (until day 21 of the assay). In studies reported by Liu et al., [24] it was found that soil microbial functional diversity and the capacity of soil microbial communities to utilise substrates were sensitive to sulfamethoxazole and chlortetracycline. Similarly, in the triclosan ecotoxicity assessment of soil microbiota, Ramires et al. [25] state that this antimicrobial agent inhibited the consumption pattern of carboxylic acids. In the study of the influence of tetracycline presence from cow manure on soil microbiota, Chessa et al. [26] evidenced that tetracycline only transiently influenced the microbiota abundance and functions.

As soil microbiome could be changed in structure, abundance, or metabolic activity once exposed to different pharmaceuticals, this could result in changes in key ecological processes of soil. At the best of our knowledge, there is at present minor information on how the presence of NSAIDs could shape rhizosphere microbiome structure, abundance and metabolic activity. Moreover, there is no information on how the presence of certain NSAIDs influence or not the secondary metabolites profile produced by rhizosphere microbiome. The main aim of this study was to identify if the presence of common NSAIDs such as ibuprofen, diclofenac and ketoprofen could shape *Lycopersicon esculentum* rhizosphere microbiota (i) abundance and phenotypic structure, (ii) metabolic activity, and (iii) secondary metabolites profile.

## 2. Materials and Methods

### 2.1. Experimental Set-Up

Pharmaceuticals such as ibuprofen (C_13_H_19_O_2_), ketoprofen (C_16_H_14_O_3_) and diclofenac (C_14_H_11_Cl_2_NO_2_), belonging to the class of non-steroidal anti-inflammatory drugs, were selected for our study. The selection of these NSAIDs was based on their increased consumption and widespread occurrence in environment.

Argic phaeozem soil samples (0–40 cm), collected in April 2020 from Cojocna, Cluj County was used in this study. The main physical chemical properties of the soil samples are listed in Table 1.

**Figure 1:**
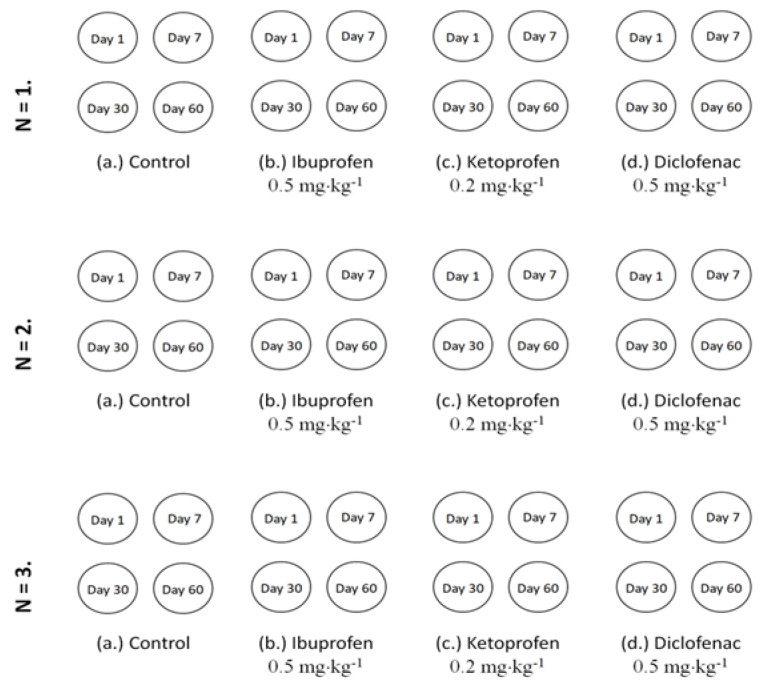
Schematic diagram of experiment set-up.

All soil samples were tested to be free of studied NSAIDs according to the method described by Kovacs et al. [27]. The samples were artificially contaminated individually with each pharmaceutical in part (0.5 mg·kg^−1^ ibuprofen, 0.2 mg·kg^−1^ ketoprofen, 0.7 mg·kg^−1^ diclofenac) as previously described [27]. For each rhizosphere soil sampling period, individual pots in triplicate were prepared for each soil type vs. pharmaceutical as presented in the schematic diagram of the experiment set-up (Figure 1). Fourteen-days-old tomato seeds (*Lycopersicon esculentum*) were planted in contaminated soil pots prepared one day before and allowed for development in a laboratory climate chamber with the following day–night cycle conditions: day—14 h of light, 25 °C; night—10 h of darkness, 18 °C. The soil water content was adjusted to assure a 58% water holding capacity (WHC) during the study.

### 2.2. Rhizosphere Microbiota Analysis through PLFA Approach

The rhizosphere soil microbiota phenotypic structure and abundance assessment was performed considering the phospholipids-derived fatty acids (PLFA) gas chromatographic analysis. The rhizosphere soils were sampled from pots contaminated with pharmaceuticals (Figure 1) after very short-term exposure (day 1), short-term exposure (day 7), mid-term exposure (day 30) and long-term exposure (day 60).

The extraction of PLFA was performed on 1 g of freeze-dried (Labconco FreeZone 6 freeze-dry system, Kansas, MO, USA) soil according to the method described by Bligh and Dyer [28] and modified by Frostegard et al. [29]. The lipids were fractionated into phospholipids, glycolipids and neutral lipids using a silicic acid column (500 mg, Phenomenex, Torrance, CA, USA). After a mild alkaline methanolysis, 150 µL of extracts containing the fatty acids methyl esters was injected into a gas chromatograph with flame ionization detector (7890A GC-FID, Agilent Technologies, Santa Clara, CA, USA). The fatty acids methyl esters separation was obtained using a 5% phenyl-methylpolysiloxane column (HP-Ultra 2, J&W Scientific, Folsom, CA, USA) with the following properties: 25 mm × 0.2 mm id., 0.33 µm film thickness. The PLFAD1 method from the MIDI Sherlock^TM^ Microbial Identification System (Microbial ID, Inc., Newark, DE, USA) was used for the phospholipids-derived fatty acids separation. The gas chromatograph operation parameters are listed in Table 2.

For the interpretation of the phospholipids-derived fatty acids data, bacterial fatty acid standards and software from MIDI Sherlock^TM^ Microbial Identification System (Microbial ID, Inc., Newark, DE, USA) was used. Saprotrophic fungi were identified using 18:2ω6c PLFA biomarker [29], and ectomycorrhizal fungi with 18:2ω9c PLFA biomarker [30]. PLFA biomarkers such as 18:2ω6c and 18:3ω3 were used for nitrogen-reducing bacteria identification [31], and 17:1ω7c, 10Me16:0, 17:1ω6, 15:1, i17:1ω7c, cy18:0ω7.8, i15:1ω7c and i19:1ω7c markers were used for sulphur-reducing bacteria identification [32,33].

### 2.3. Rhizosphere Microbiota Responses Evaluation

The rhizosphere microbiota response to ibuprofen, diclofenac and ketoprofen was evaluated considering community-level physiological profile (CLPP) and emitted volatile organic compounds (VOCs). The sampling of the rhizosphere soil samples from the contaminated pots was performed according to the schematic diagram presented in Figure 1.

#### 2.3.1. Community-Level Physiological Profile (CLPP)

The assessment of the metabolic activity of rhizosphere soil microbiota after exposure to NSAIDs was performed using Biolog EcoPlate^TM^ containing 31 different carbon sources. At each established sampling period, 2 g of rhizosphere soil samples were used to extract the microbiota with a 10 mL PBS solution. The extraction was allowed for 30 min through continuous mechanical shaking (LaboShake, Gerhardt Analytical System, Konigswinter, Germany), followed by 1 h of rest. The mixture of soil suspension and supernatant was subjected to sonication and centrifugation as described by Lindahl and Bakken [34] until we obtained the final soil lixiviate. The soil particles from the obtained lixiviates were removed through low-speed centrifugation (1000 rpm for 1 min, LMC-3000 centrifuge, GrantBio, Riga, Latvia). From this final solution, 150 µL was added to each well of EcoPlate and incubated under dark conditions at 25 °C for 3 days (LabCompanion, Billerica, MA, USA). The optical density (OD) of each well was measured at λ = 590 nm using an SpectraMax iD3 Microplate Reader (Molecular Devices, San Jose, CA, USA) and SoftMax Pro7 software (Molecular Devices) just after inoculation and once a day during the incubation period.

#### 2.3.2. Emitted VOCs

The rhizosphere soil-emitted volatile organic compounds content was determined through headspace-solid phase microextraction sampling using 85 µm polyacrylate fibre (Supelco Inc., Bellefonte, PA, USA). An amount of 1 g of soil samples was diluted with 2 mL of PBS solution in 20 mL headspace glass vials (Agilent Technologies). The headspace vials were tightly capped with Teflon-faced rubber liner caps and subjected for incubation for 72 h in dark at 25 °C. After the incubation period, the vials were equilibrated for 30 min at 60 °C using a TriPlus RSH autosampler (Thermo Scientific, Austin, TX, USA). The SPME fibre after activation in the SSL injector was exposed and maintained in the vial headspace surface for 15 min. The volatile profile analysis was performed on GC-MS/MS (Trace 1310, TSQ 9000, Thermo Scientific, Austin, TX, USA) with electron impact ionization (70 eV ionization energy). The separation was performed on Agilent HP-5MS capillary column (30 m × 0.25 mm, 0.25 µm) using helium as carrier gas with 1.2 mL·min^−1^ flow. SPME fibre was injected into the GC injection port and adsorbed volatiles in the fibre were desorbed onto the column at 250 °C for 5 min in splitless mode. The volatile organic compounds were identified by comparison of their mass spectra with compounds corresponding to mass spectra library (NIST/EPA/NIH, Chromeleon 7.2 CDS Software, Thermo Scientific, Austin, TX, USA).The identified volatile organic compounds were expressed in percentages as a normalised amount of each volatile organic compound resulted after the division of peak areas of identified volatile organic compounds by total peak area of all identified volatile organic compounds.

### 2.4. Statistical Interpretation of Data

The differences in rhizosphere soil microbial community composition were investigated by principal component analysis (PCA) using Statistica 10 software (StatSoft, Hamburg, Germany). For the statistical analysis, the OD of each well after inoculation was subtracted from the OD after each measurement period during the incubation. The averages and standard deviations corresponding to each carbon source were determined as samples were analysed in triplicate, since Biolog EcoPlates contain three replicates of each carbon source. The average well colour development (AWCD), Richness (S), Shannon’s diversity index (H’) and Shannon’s evenness index (E) were determined according to the formulas presented by Sofo and Ricciuti [35]. All these parameters were calculated separately for all incubation times. For Richness, a 0.25 value for optical density (OD) was set as the threshold for a positive response [35]. ANOVA was conducted to assess the effect of the studied pharmaceuticals on the community-level utilisation of carbon sources. The assumption of the homogeneity of variance and the test for normality of distributions were verified applying Levene’s test and Shapiro–Wilk’s test using Statistica 10 software version (StatSoft, Germany). A level of *p* = 0.05 was considered to assume statistical significance. The UpSetR diagrams were performed according to Khan and Mathelier [36].

## 3. Results and Discussions

### 3.1. Rhizosphere Microbiota Abundance Changes with Contamination of NSAIDs

**Figure 2:**
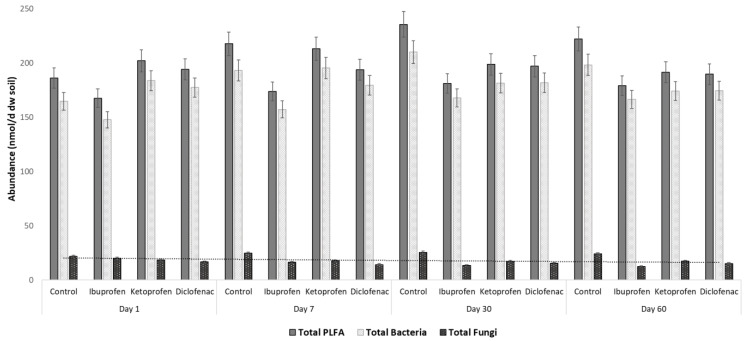
Soil microbiota abundance variation in rhizosphere soil during assay period.

The structure and abundance of microbiota were monitored in *Lycopersicon esculentum* rhizosphere soils with and without the artificial contamination with commonly consumed NSAIDs during a 60-day assay period. The total microbial biomass was expressed as the sum of PLFAs with concentrations in control samples (without contamination) and those contaminated ranged between 165.6 and 240 nmol·g^−1^ dry weight soil during the assay (Figure 2). The control rhizosphere soil recorded higher values of PLFA (*p* < 0.05) with concentration ranges of 184.8–240 nmol·g^−1^ dry weight soil. In soils contaminated with NSAIDs, the microbial biomass in *Lycopersicon esculentum* rhizosphere soils has the following pattern: 187.9–215.4 (ketoprofen contamination) > 186.7–201.2 (diclofenac contamination) > 165.6–182.7 (ibuprofen contamination) nmol·g^−1^ dry weight soil, respectively (Figure 2).

**Figure 3:**
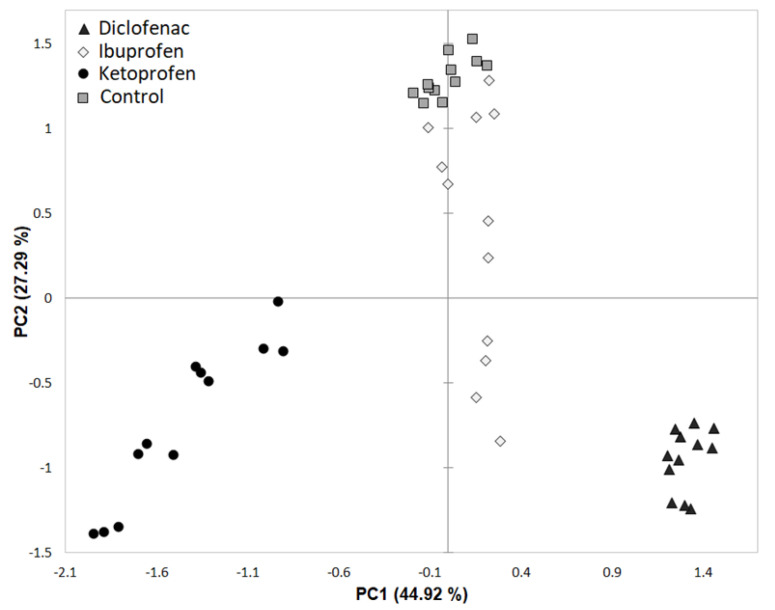
Principal component analysis (PCA) of rhizosphere PLFAs from control samples and those contaminated with different NSAIDs.

The PCA of the 48 PLFAs data (Figure 3) indicated that rhizosphere soil microbial community abundance was markedly affected by soil contamination with NSAIDs, but poor differentiation between control and contamination with ibuprofen was observed, indicated by their closest scores along the first principal component (PC1) and second principal component (PC2). The first two components, PC1 and PC2 explained 44.92% and 27.29% of the total variance in PLFAs abundance. The PC1 axis differentiated contamination with diclofenac and ketoprofen but did not differentiate controls by contamination with ibuprofen, whereas the PC2 axis did not differentiate well control samples by contamination with specific NSAIDs.

### 3.2. Rhizosphere Microbiota Community Structure Changes in Time with Contamination of NSAIDs

Rhizosphere microbiota structure abundance differed through the assay sampling periods in all studied cases. Starting from day one until day thirty of the assay, an increasing tendency was observed, followed by a stabilization until day sixty of the assay (Figure 2).

*Lycopersicon esculentum* rhizosphere soils presented a bacterial dominance (Table 3) in all studied assays. The ratios of fungi to bacteria (F/B), Gram-negative bacteria to Gram-positive bacteria (G−/G+), aerobes bacteria to anaerobes bacteria (AerB/AnB) and ectomycorrhizal fungi/saprotrophic fungi (Ecto/Sapro) are presented in Table 3.

According to that, it was observed that among bacterial communities, a higher dominance was observed in the case of Gram-negative and aerobic bacteria ones. The average amount of fungal/bacterial ratio among the contaminated soils with specific NSAIDs and the period of exposure revealed the following pattern: control > contamination with ibuprofen > contamination with ketoprofen > contamination with diclofenac. Compared with control, the average value of the ratio of Gram-negative/Gram positive bacteria was higher in the rhizosphere soil contaminated with ibuprofen and lower in the case of diclofenac contamination (Table 3). Similarly, the ratio of aerobic/anaerobic bacteria presented an increasing tendency compared with that of control, with the following pattern: control < contamination with ibuprofen < contamination with ketoprofen < contamination with diclofenac (Table 3).

**Figure 4:**
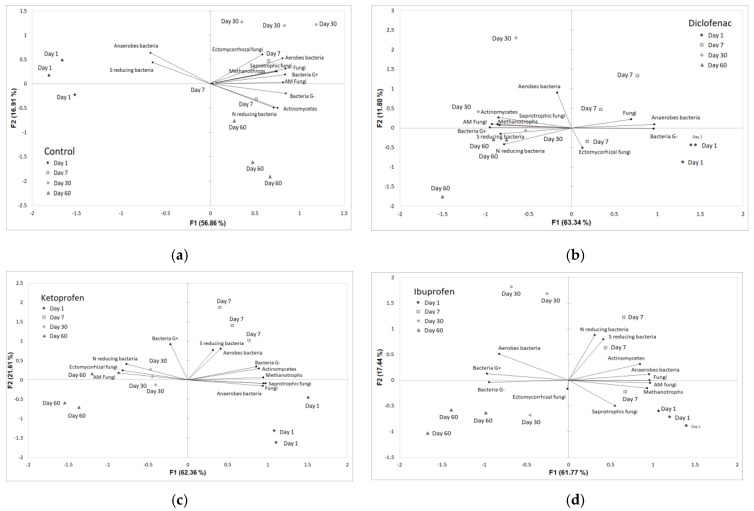
Principal component analysis (PCA) of rhizosphere microbial community composition evolution in time for each contamination assay: (**a**) control, (**b**) diclofenac, (**c**) ketoprofen, (**d**) ibuprofen.

The principal component analysis was used as a data reduction strategy to infer correlations between rhizosphere microbiota community structure abundance evolution in time for each contamination experiment (Figure 4). The first and second components of the principal coordinate accounted for 56.86% and 19.91% of the total variance of the microbiota structure abundance in time for no contamination (control, Figure 4a), 63.34% and 11.8% for diclofenac contamination (Figure 4b), 62.36% and 21.61% for ketoprofen contamination (Figure 4c) and 61.77% and 17.44% for ibuprofen contamination (Figure 4d).

In the control assay, aerobes and sulphur-reducing bacteria PLFAs abundance was differentiated by the first axes with 56.86% by the rest of rhizosphere microbiota community PLFAs in day 1. The second axes differentiated Gram negative, nitrogen-reducing bacteria and actinomycetes by the rest of the rhizosphere community with 16.91% (Figure 4a). For diclofenac assay, fungi, anaerobes bacteria, Gram-negative bacteria and ectomycorrhizal fungi clearly differentiated by the rest of community through first axes (63.34%) in day 1 and 7 of the assay (Figure 4b). Through the contamination with ketoprofen differentiation within sulphur-reducing bacteria, aerobes bacteria, Gram-negative bacteria, actinomycetes, methanotrophs bacteria, anaerobes bacteria, saprotrophic fungi and fungi were observed through the first axes in day 1 and 7 of the assay with 63.36% (Figure 4c). In the assay of the impact of ibuprofen on rhizosphere microbiota community structure abundance, the first axes of PCA differentiated with 61.77% nitrogen- and sulphur-reducing bacteria, actinomycetes, anaerobes bacteria, fungi, arbuscular mycorrhizal fungi, saprotrophic fungi and methanotrophs bacteria in day 1 and 7 of assay by the rest of the community (Figure 4d).

### 3.3. Community-Level Physiological Profile Changes in Time with Contamination of Nsaids

Rhizosphere microbiota potential metabolic activity can be inferred by the AWCD where the highest value of AWCD indicates a higher metabolic activity of microorganisms (Table 4). We performed the analysis after very short-term exposure (day 1), short-term exposure (day 7), midterm exposure (day 30) and long-term exposure (day 60) at studied NSAIDs. Based on the indices of the carbon sources consumption base metabolic diversity, the highest score for functional richness (S) was determined in control samples (ranged between 13 to 20), followed by samples contaminated with diclofenac (Table 4), although a minor differentiation was obtained for Shannon’s diversity index (H’) and Shannon’s evenness index (E) among control and contaminated samples.

Carbon source consumption increased with the time of assay in the case of control samples and those contaminated with diclofenac. This suggests that rhizosphere microbiota under contamination with diclofenac consume a higher amount of carbon, but they do not consume a larger variety of its sources. In the case of contamination with ibuprofen and ketoprofen, the consumption of carbon source presents a decreasing tendency after day 30 of the assay (Table 4). Compared with PLFA data, we supposed that rhizosphere communities are resilient at broad but not fine phenotypic levels: the abundance of Gram-negative bacteria, anaerobes bacteria, actinomycetes and arbuscular mycorrhizal fungi abundance under contamination with diclofenac decreased with approximately 35.5, 54.7, 53 and 49.4%, respectively, compared with control.

**Figure 5:**
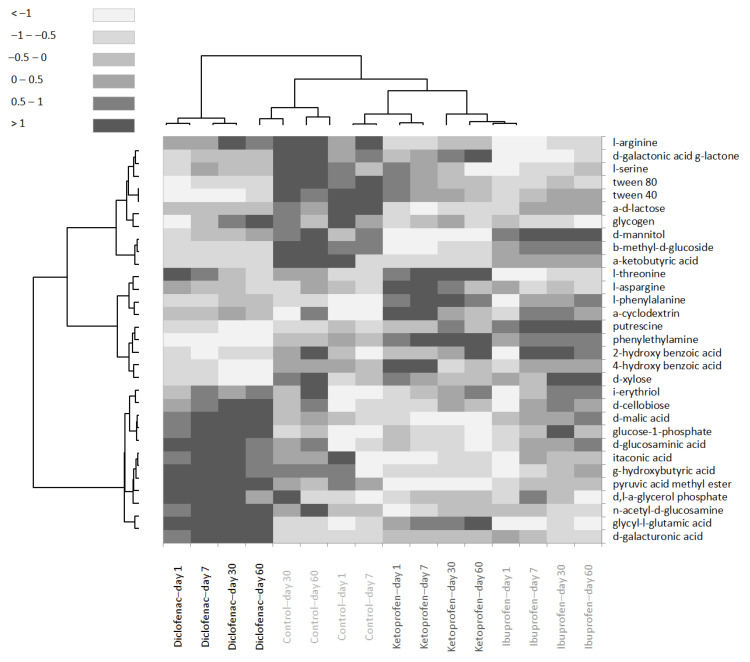
Heat map of rhizosphere microbial community-level physiological profile changes in time for each contamination assay.

The heat map analysis (Figure 5) showed that in control samples, a poor consumption was determined after the very short-term period of the assay for glycyl-glutamic acid, d-glucosaminic acid, glucose-1-phosphate, d-cellobiose, i-erythritol, α-cyclodextrin and l-phenylalanine, while after the long-term assay period, an increase in tetrazolium reduction was detected for n-acetyl-d-glucosamine, d-glucosaminic acid, d-cellobiose, i-erythritol, 2-hydroxy benzoic acid, α-cyclodextrin and d-mannitol. The relative constant consumption during the entire assay period was registered in control samples for carbon sources as carboxylic acids (γ-hydroxybutyric acid, d-malic acid, α-ketobutyric acid), carbohydrates (β-methyl-d-glucoside and d-l-lactose) and others, such as 4-hydroxy benzoic acid, phenylethylamine and l-arginine.

Towards the control samples, under exposure with diclofenac, a decreased tendency in time was observed for phenolic compounds (F = 4.924, *p* = 0.004) and amines (F = 18.5, *p* < 0.0001), while carbohydrates (F = 4.568, *p* = 0.043) and carboxylic acids (F = 33.888, *p* < 0.0001) increased until the end of the assay period. In rhizosphere soil contaminated with ibuprofen, a decrease in consumption for d-galacturonic acid and n-acetyl d-glucosamine was recorded. Compared with control, an increase in consumption of carboxylic acids (F = 10.04, *p* < 0.001) and amino acids (F = 23.504, *p* < 0.0001) was observed at the end of the assay. No influence on the tetrazolium reduction rate was determined for carbon substrates such as pyruvic acid methyl ester, 4-hydroxybenzoic acid, phenylethylamine, putrescine, α-keto butyric acid, α-d-lactose and d-mannitol. In the assays of exposure with ketoprofen, a poor consumption was recorded in the case of pyruvic acid methyl ester and d-mannitol substrates. Towards the control sample assays, an increase in amino acids (glycyl-l-glutamic acid, l-threonine, l-arginine) consumption was observed until the end of the assay (F = 13.009, *p* < 0.0001). The polymer substrates consumption decreased in time compared with control samples (F = 20.547, *p* = 0.012).

Overall, the rhizosphere communities in the presence of different NSAIDs changed the carbon consumption for each substrate classes. In soils exposed to ketoprofen and diclofenac, a significant decrease in the utilisation of carbon substrates was observed compared with soil exposed to ibuprofen or the control sample (Table 4, Figure 5).

### 3.4. Microbiota Emitted Volatile Organic Compounds Changes in Time with Contamination of Nsaids

We performed a VOCs analysis by SPME-GC-MS in all control and contaminated rhizosphere soils during the exposure assay. Volatile compounds belonging to alcohol, aromatic compounds, ketone, terpene, organic acids, aldehyde, sulphur compounds, esters, alkane, nitrogen compounds, alkene and furans were detected in the rhizosphere soil samples. Terpene, ketone, alcohol, aromatic compounds, organic acids and alkane were the most abundant compound classes (Table 5). In the control samples, the highest alcohol compound was hexan-1-ol followed by 1-octen-3-ol with the average amount during the assay periods of 2.87 and 2.64%, respectively. Benzaldehyde was the highest aromatic compound identified in control samples and those contaminated with diclofenac (3.38%) and ibuprofen (6.61%). In the case of ketoprofen contamination, phenol was the highest aromatic compound released by rhizosphere microbiota (3.25%). Among ketones, the prevalent compounds were decane-2-one (3.41%, diclofenac contamination), octan-3-one (control and ketoprofen contamination, 2.84%) and decan-2-one (ibuprofen contamination, 3.34%). Terpineol was determined in similar amounts in the case of the control sample, as well in the case of contamination with ketoprofen (5.55%). Compounds such as geranyl acetone (5.84%) and germacradien-1l-ol (4.89%) were the most abundantly released terpenes by rhizosphere microbiota under contamination with ibuprofen and diclofenac, respectively. Butanoic acid was the most prevalent organic acid in all studied rhizosphere soils, with the highest amount identified in rhizosphere soils under contamination with diclofenac (4.32%), followed by control and ketoprofen-contaminated soil samples (3.44%) and those contaminated with ibuprofen (2.84%).

**Figure 6:**
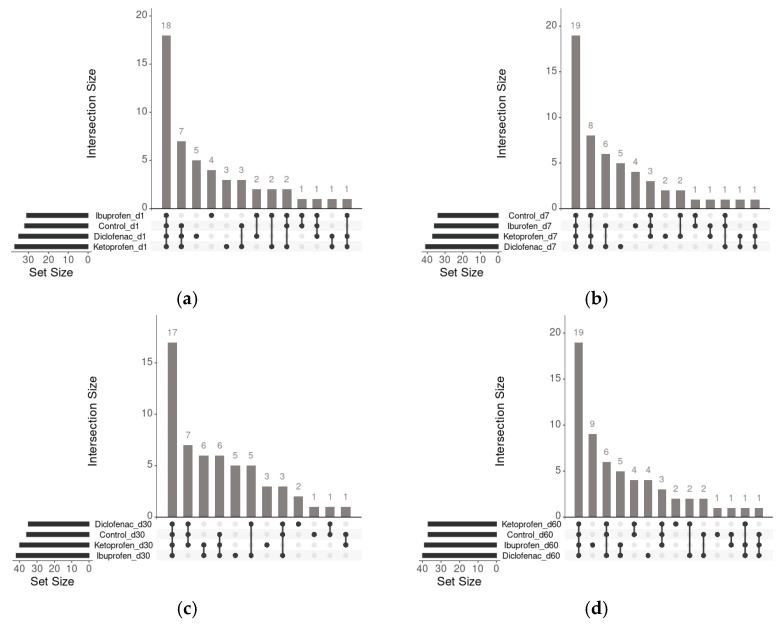
UpSet plot of interactions and the amounts of similar and different volatile organic compounds released by rhizosphere microbial community composition evolution in time for each contamination assay. (**a**) after one day assay; (**b**) after 7 day assay; (**c**) after 30 day assay; (**d**) after 60 day assay.

To identify volatile organic compound-specific or shared regulations, we compared the sets of upregulated volatile organic compounds obtained for each exposure case and time of assays (Figure 6).

Between 16 and 11 upregulated volatile organic compounds responded specifically to the presence of NSAIDs. Among the remaining volatile organic compounds that were upregulated by at least two different pharmaceutical compounds, the highest intersection size was found to increase once with the duration of the assay (19 volatile organic compounds representing 37% of the total number of upregulated volatile organic compounds in rhizosphere soils after 60 days of assay, Figure 6d). Interactions varied in time with assay duration, volatile organic compounds that were upregulated by control and at least two different NSAIDs. The highest size was found after midterm exposure (day 30, Figure 6c) and short-term exposure (day 7, Figure 6b) of the assay. The similarities of volatile organic compounds in assays at very short-term exposure (day 1) were found for two upregulated volatile organic compounds when rhizosphere microbiota were exposed to ibuprofen and ketoprofen and two other upregulated volatile organic compounds were found when the exposure was for ibuprofen and diclofenac (Figure 6a). In the case of the short-term exposure assay (day 7), similarities of the volatile organic compounds were found for six upregulated volatile organic compounds when rhizosphere microbiota were exposed to ibuprofen and diclofenac (Figure 6b), five upregulated volatile organic compounds when rhizosphere microbiota were exposed to ibuprofen and diclofenac during the midterm exposure assay, and six upregulated volatile organic compounds when rhizosphere microbiota were exposed to ibuprofen and ketoprofen (Figure 6a).

There is currently poor information related to the effects of commonly reported NSAIDs on the rhizosphere microbiome abundance and metabolic activity. Most studies refer to the impact of antibiotics on bulk soil microbiome. Therefore, it was difficult to compare our results with those obtained by other authors. In general, the reported data showed differentiated the effects of pharmaceuticals on bulk soil microorganisms, the data differing for each pharmaceutical in part, the concentration of pharmaceuticals and time of exposure [37,38]. In addition, most studies involved assays on isolated microorganisms [39,40,41,42] and not on rhizosphere microbiome. However, these data often showed changes in strain vitality or their enzymatic activity. The obtained data in the study performed on *Pseudomonas sp.*, by Aleanizy et al. [42] showed a reduction in abundance, pyocyanin production and specific enzymatic activity (protease, DNase) after exposure to antibiotics such as azithromycin, piperacillin/tazobactam and cefepime. Dai et al. [39] have shown in their study that *Pseudomonas aeruginosa* biofilm formation and adherence acidity significantly reduced after exposure to ibuprofen. Similarly, Oliviera et al. [41] reposted that *Staphylococcus aureus* growth in planktonic and biofilm states was controlled by ibuprofen exposure in different concentrations.

In this study, it was observed that the *Lycopersicon esculentum* rhizosphere microbiome abundance decreased especially under exposure to ibuprofen and diclofenac. Changes in the phenotypic structure was also observed in the experimental data, especially in the case of fungal/bacteria, Gram-positive/Gram-negative bacteria and aerobic/anaerobic bacteria ratios. These data followed those reported by Cycon et al. [10], Paje et al. [41] and Dastidar et al. [43]. Considering the rhizosphere microbiome emitted volatile organic compounds, a differentiated pattern was observed in time, depending also on the NSAIDs used in the exposure experiment. In the case of diclofenac exposure assessment, our data reveal an increasing tendency with time in the case of specific alcohol compounds (2-methyl-butan-1-ol, hexan-1-ol, and 1-octen-3-ol), aromatic compounds (benzaldehyde, 1-methoxy-4-methylbenzene, and phenol) and germacradien-1l-ol. In the case of alcohol compounds, a similar pattern was observed even when the contamination was performed with ketoprofen. The remaining volatile organic compounds either decreased during the assay time or presented an increasing tendency during the first half period of the assay and decreased after that. Comparing these patterns with those reported in the literature, we found that Zhou et al. [44] in their study on extracellular polymeric substances once observed an increase with an exposure to ibuprofen. However, in all contamination assessments, different patterns of volatile organic compounds emission were founded. Because volatile organic compounds are essential for above and belowground diversity, it becomes important to understand how these are affected by the presence of certain pharmaceuticals. Similarly, rhizosphere microbiome carbon consumption pattern differed also once with contamination to studied NSAIDs. Towards the control samples, in soils exposed to ketoprofen and diclofenac carbon substrates, the consumption was lower. Minor changes were observed in the case of exposure to ibuprofen.

Pharmaceuticals are essential for human and animal health treatment and maintenance. As soil microbiota abundance, structure and functioning could be modified in the presence of pharmaceuticals, it becomes urgent to identify solutions to limit the spread of pharmaceutical residues, antibiotic-resistant bacteria and genes in environmental compartments. One way is by improving wastewater treatment plant efficiency to remove such micropollutants. However, better knowledge is required on the fate of pharmaceuticals in the environment and on the underlying processes, on interactions within biota and induced changes.

## 4. Conclusions

In this study, the impact of commonly reported NSAIDs in the environment on Lycopersicon esculentum rhizosphere microbiota was investigated. The obtained results clearly illustrate that the presence of drugs could shape rhizosphere microbiota abundance and phenotypic structure. Comparing the data with those of control, it was observed that especially under exposure to diclofenac and ibuprofen, the rhizosphere microbiota abundance decreased with approximately 10–20%. Additionally, microbiota metabolic activity modified in contact with NSAIDs (e.g., increase of carbon consumption rate with 8% under exposure to diclofenac). Such changes make us suppose that there is a probability to also change ecosystem services provided by rhizosphere microbiota. Therefore, to protect soil ecosystem services mediated by rhizosphere microbiome, the presence of potential pharmaceuticals should be considered. On this basis, it will be possible not only to assess the presence of NSAIDS or their impact on microorganisms, but also to look for links between the changes of microbiota community functioning and soil ecosystem services that they mediate.

## Figures and Tables

**Table 1:** Average values (*n* = 12) of physical chemical properties of the studied soil samples.

Soil Property	Argic Phaeozem
Clay	27.2 ± 0.94
Sand	16.1 ± 0.20
Silt	56.7 ± 1.37
Texture	Silty Clay Loam
Moisture (cm^3^/cm^3^)	0.344 ± 0.01
Soil temperature (°C)	10.4 ± 0.09
Organic carbon (%)	6.2 ± 0.12
pH	5.9 ± 0.09

**Table 2:** GC-FID operation parameters for phospholipids-derived fatty acids analysis from rhizosphere soils.

Parameter	Conditions
Inlet temperature	280 °C
Split mode	40:1
Oven temperature program	170 °C, increase with 28 °C·min^−1^ until 288 °C, followed by a new increase with 60 °C·min^−1^ until 310 °C. This final temperature was maintained constant for 1.25 min
Flow	1.2 mL·min^−1^
Detector temperature	300 °C

**Table 3:** Microbiota phenotypic structure components ratio variation among contamination.

NSAIDs	Day	Fungi/ Bacteria	Gram (−)/ Gram (+)	Aerobes/ Anaerobes	Ectomycorrhizal/ Saprotrophic
Control	1	0.131	2.585	2.157	0.695
	7	0.128	2.422	3.286	0.496
	30	0.122	2.151	3.090	0.669
	60	0.121	2.518	3.274	0.614
Ibuprofen	1	0.135	4.926	2.111	0.702
	7	0.104	3.382	2.351	0.916
	30	0.080	3.165	3.211	0.849
	60	0.075	3.157	4.820	0.835
Ketoprofen	1	0.100	2.379	2.805	1.033
	7	0.090	1.887	3.747	1.441
	30	0.095	1.950	3.977	1.594
	60	0.099	1.946	4.386	2.360
Diclofenac	1	0.094	2.764	4.063	0.722
	7	0.079	2.113	5.825	0.564
	30	0.085	1.604	6.740	0.555
	60	0.087	1.359	9.143	0.499

**Table 4:** Average well colour development (AWCD), Richness (S), Shannon’s diversity index (H’) and Shannon’s evenness index (E) variation among contamination.

NSAIDs	Day	AWCD	S	H	E
Control	1	0.26 ± 0.011	16.67 ± 0.577	3.20 ± 0.027	1.14 ± 0.006
	7	0.24 ± 0.007	13.33 ± 1.155	3.04 ± 0.016	1.17 ± 0.035
	30	0.30 ± 0.002	17 ± 0.00	3.21 ± 0.006	1.13 ± 0.002
	60	0.32 ± 0.003	20.33 ± 1.155	3.21 ± 0.004	1.07 ± 0.022
Ibuprofen	1	0.19 ± 0.003	9 ± 0.00	3.03 ± 0.02	1.38 ± 0.009
	7	0.25 ± 0.006	11.33 ± 0.577	3.16 ± 0.061	1.30 ± 0.011
	30	0.27 ± 0.005	13.00 ± 1.732	3.22 ± 0.012	1.26 ± 0.057
	60	0.26 ± 0.002	12.00 ± 0.00	3.20 ± 0.002	1.29 ± 0.001
Ketoprofen	1	0.24 ± 0.003	14.67 ± 0.577	3.15 ± 0.008	1.17 ± 0.019
	7	0.25 ± 0.004	15.0 ± 0.00	3.13 ± 0.026	1.15 ±0.010
	30	0.24 ± 0.002	13.0 ± 0.00	3.16 ± 0.003	1.23 ± 0.001
	60	0.23 ± 0.001	11.33 ± 0.577	3.12 ± 0.003	1.29 ± 0.025
Diclofenac	1	0.25 ± 0.009	13.0 ± 0.00	3.14 ± 0.020	1.23 ± 0.072
	7	0.27 ± 0.006	13.67 ± 0.577	3.04 ± 0.108	1.16 ± 0.037
	30	0.28 ± 0.003	15.33 ± 1.155	3.13 ± 0.010	1.15 ± 0.029
	60	0.27 ± 0.006	15.67 ± 1.155	3.10 ± 0.020	1.13 ± 0.025

**Table 5:** Distribution of volatile organic compounds (%) produced in rhizosphere soil samples (control and NSAIDs contaminated).

Volatile Organic Compounds	Control	Ketoprofen	Ibuprofen	Diclofenac
Alcohol	12	13	18	12
Aromatic compounds	11	11	15	11
Ketone	13	14	11	12
Terpene	18	18	14	12
Organic acids	12	12	9	15
Aldehyde	6	8	6	8
Sulphur compounds	2	1	6	5
Ester	4	4	5	5
Alkane	12	11	10	11
Nitrogen compounds	5	5	4	3
Alkene	4	3	2	6
Furans	1	0	0	0

## Data Availability

The data that support the fundings of this study are available on request from the corresponding author (KED).
